# Commonly Applied Selection Criteria for Lung Cancer Screening May Have Strongly Varying Diagnostic Performance in Different Countries

**DOI:** 10.3390/cancers12103012

**Published:** 2020-10-16

**Authors:** Hermann Brenner, Agne Krilaviciute

**Affiliations:** 1 Division of Clinical Epidemiology and Aging Research, German Cancer Research Center (DKFZ), 69120 Heidelberg, Germany; a.krilaviciute@dkfz-heidelberg.de; 2 Division of Preventive Oncology, German Cancer Research Center (DKFZ) and National Center for Tumor Diseases (NCT), 69120 Heidelberg, Germany; 3 German Cancer Consortium (DKTK), German Cancer Research Center (DKFZ), 69120 Heidelberg, Germany

**Keywords:** lung cancer, screening, smoking, sensitivity, specificity

## Abstract

**Simple Summary:**

Smoking causes the majority of lung cancers. Smoking history is thus used to select individuals among whom screening for lung cancer could be the most beneficial. The aim of our study was to estimate sensitivity and specificity of pre-selection by heavy smoking in individual European countries. Due to differences in smoking histories across the countries and sexes within the countries, the sensitivities were found to be between 33 and 80% for men and between 9 and 79% for women. Corresponding specificities of heavy smoking varied between 48 and 90% (men) and 70 and 99% (women). Our results may inform the design of lung cancer screening programs in European countries and serve as benchmarks for novel alternative or complementary tests for selecting people at high risk for computed tomography-based lung cancer screening.

**Abstract:**

Lung cancer (LC) screening often focuses heavy smokers as a target for screening group. Heavy smoking can thus be regarded as an LC pre-screening test with sensitivities and specificities being different in various populations due to the differences in smoking histories. We derive here expected sensitivities and specificities of various criteria to preselect individuals for LC screening in 27 European countries with diverse smoking prevalences. Sensitivities of various heavy-smoking-based pre-screening criteria were estimated by combining sex-specific proportions of people meeting these criteria in the target population for screening with associations of heavy smoking with LC risk. Expected specificities were approximated by the proportion of individuals not meeting the heavy smoking definition. Estimated sensitivities and specificities varied widely across countries, with sensitivities being generally higher among men (range: 33–80%) than among women (range: 9–79%), and specificities being generally lower among men (range: 48–90%) than among women (range: 70–99%). Major variation in sensitivities and specificities was also seen across different pre-selection criteria for LC screening within individual countries. Our results may inform the design of LC screening programs in European countries and serve as benchmarks for novel alternative or complementary tests for selecting people at high risk for CT-based LC screening.

## 1. Introduction

Lung cancer (LC) is the leading cause of cancer-related mortality in both men and women, accounting for more than 1.7 million deaths each year globally [1]. Due to asymptomatic onset of the disease, most LC cases are detected at advanced stages when chances of cure are very limited. So far, more than 80% of LC patients still die within five years after diagnosis [2].

Major efforts have been made to lower the burden of LC mortality by effective early detection. The U.S. National Lung Screening Trial (NLST) found a 20% reduction of LC-specific mortality by low-dose computed tomography (CT) as compared to chest X-ray screening [3]. The effectiveness of low-dose CT screening was recently reconfirmed by the largest European randomized trial (NELSON study) after 10 years of follow-up (reduction in LC mortality of 24% and 33% as compared to no screening among men and women, respectively) [4]. The employed screening strategies, though, still suffer from high rates of false positive results, considerable resource requirements, and not yet fully determined potential harms, e.g., due to ionizing radiation.

The U.S. Preventive Services Task Force and Centers for Medicare and Medicaid Services recommend low-dose CT screening of heavy smokers with at least a 30 pack-year smoking history [5,6]. Organized LC screening is not yet established in Europe and selection criteria that were used in European CT screening trials differ with respect to the potential target population eligible for screening. Except from the UK Lung Cancer Screening Trial (UKLS) that identified the target group based on an individual LC risk model [7], other screening trials focused on smoking habits alone, by screening heavy smokers with a certain pack-year exposure [8,9,10,11] or heavy smokers with a defined smoking intensity over a period of time [12,13,14]. Determination of heavy smoking, as the best-established LC risk factor, could be regarded in this context as a pre-screening test to enhance the efficiency of screening by low-dose CT.

Smoking habits strongly vary between countries and between sexes within countries, which implies that the proportion of heavy smokers among people with and without LC, and hence the sensitivity and specificity of such pre-selection for LC screening, is expected to strongly vary between countries. The aim of this study was to estimate sensitivity and specificity of diagnostic performance of the various smoking eligibility criteria used in the NLST and the European LC screening trials as an LC pre-screening test in different European countries. Sensitivity in this context quantifies the proportion of preclinical LC cases that would be offered CT screening, and specificity quantifies the proportion of people without preclinical LC that would not be offered CT screening. Besides providing a basis for evaluating and comparing sex-specific diagnostic performance of alternative definitions of smoking history for each country, our estimates may serve as benchmarks of diagnostic performance of potential alternative more comprehensive risk factor models or complementary noninvasive or minimally invasive biomarker-based pre-screening tests, which are intensively searched for globally, such as breath tests [15,16,17,18], or blood-based tests, e.g., based on micro-RNA signatures [19,20,21,22].

## 2. Results

### 2.1. Smoking Prevalence in European Countries

**Figure 1:**
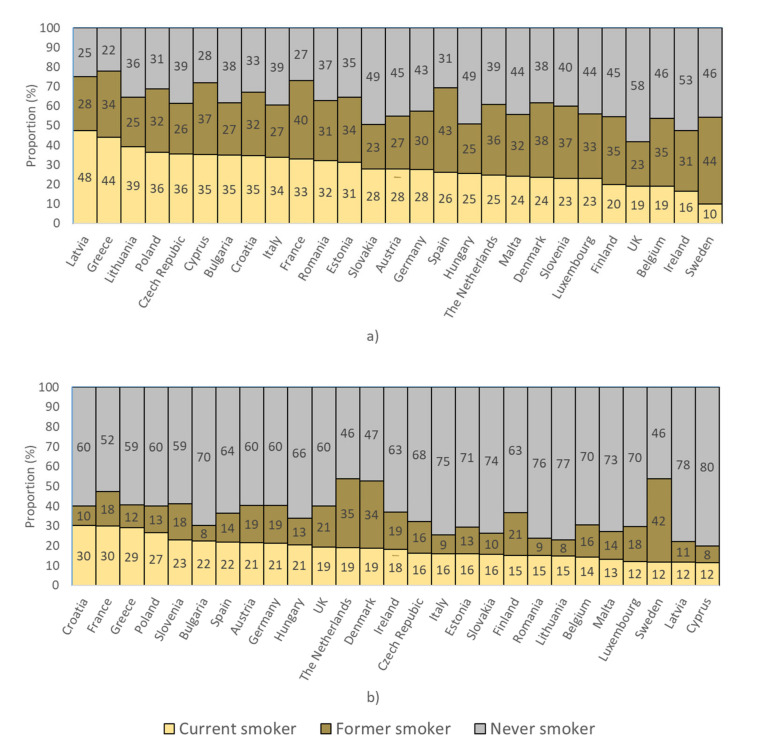
Proportion of individuals aged 50–74 years in the Eurobarometer database classified according to their smoking status among (**a**) men and (**b**) women.

Figure 1 shows the sex-specific proportions of current, former and never smokers by country. Smoking prevalences varied strongly between countries, and the proportions of current and former smokers were substantially higher for men than for women within most countries. Prevalence of current smoking ranged from 48% among Latvian men to 10% among men from Sweden, and the proportion of never smokers ranged from 22% among Greek men to 80% among women from Cyprus.

### 2.2. Populations Eligible for Screening

Appendix A and Appendix B show the proportions of men and women within the age ranges included in the various trials that would meet eligibility for LC screening based on smoking history. These proportions ranged from 10% (Sweden, NLST criteria) to 52% (Greece, NLST criteria) among men, and from 1% (Lithuania, NLST criteria) to 30% (Greece, ITALUNG criteria) among women. Current smokers accounted for the majority of eligible men and women in all countries except Sweden and Denmark, where current and former smokers accounted for approximately equal proportions of eligible men and women, respectively.

### 2.3. Estimates of Sensitivity and Specificity for Smoking History and Age

**Figure 2:**
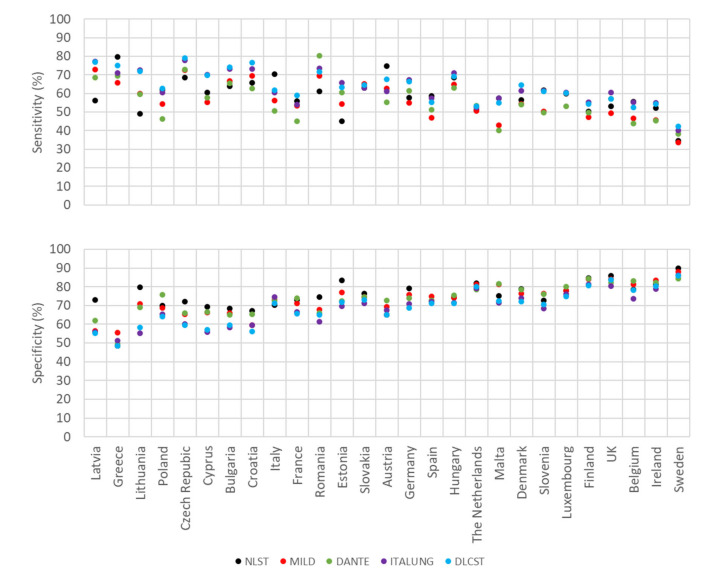
Estimates of sensitivity and specificity of various smoking-based criteria as a pre-screening test for lung cancer in European countries among men.

**Figure 3:**
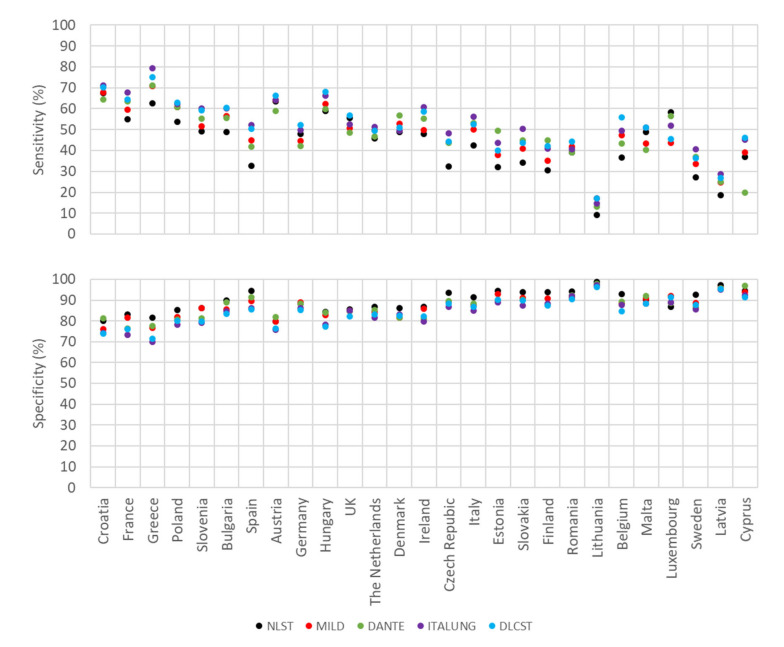
Estimates of sensitivity and specificity of various smoking-based criteria as a pre-screening test for lung cancer in European countries among women.

Figure 2 and Figure 3 show estimates of sensitivity and specificity for the various smoking definitions when applied as a pre-screening test in European countries among men and women, respectively. Within each figure, countries are ordered, from highest to lowest, by the prevalence of current smoking. Appendix C and Appendix D list corresponding exact values for each country.

Among men (Figure 2, Appendix C), estimated sensitivities varied between 33% in Sweden (MILD criteria) and 80% in Romania (DANTE criteria) and specificities were between 48% in Greece and 90% in Sweden (both NLST criteria). Countries with higher sensitivities tended to exhibit lower specificities, and vice versa. The lowest sensitivities (≤42%) and highest specificities (≥86%) were seen in Sweden for all LC screening criteria. Differences in sensitivity estimates within each country are seen due to different populations being eligible for screening for each trial criteria, where differences in sensitivities larger or equal to 20 percent units were observed for Latvia, Lithuania and Estonia.

Among women (Figure 3, Appendix D), estimated sensitivities varied between 9% in Lithuania (NLST criteria) and 79% in Greece (ITALUNG criteria), and specificities ranged from 70% in Greece (ITALUNG criteria) to 99% in Lithuania (NLST criteria). Again, countries with higher sensitivities tended to exhibit lower specificities, and vice versa. The lowest sensitivities (≤17%) and highest specificities (≥96%) were seen in Lithuania for all LC screening criteria. For some countries, major variation in estimated sensitivity close to (Spain, Belgium) or above (Cyprus) 20 percent units was seen between the various criteria for heavy smoking used in the different trials, whereas specificity estimates were generally quite close across the various criteria.

## 3. Discussion

In this study, we derived sex-specific estimates of sensitivity and specificity of various heavy smoking criteria used in LC screening trials as a pre-screening test for LC detection for 27 European countries. We demonstrated that the same criterion may lead to strongly varying sensitivities and specificities when applied to European countries with strongly varying smoking histories, with sensitivities generally expected to be higher and specificities generally expected to be lower in countries with higher smoking prevalences than in countries with lower smoking prevalences and among men than among women. Some major variation was also seen in sex-specific estimates of sensitivity of the various heavy smoking criteria within countries.

Despite the very strong association of smoking with LC, the diagnostic performance of various definitions of smoking as a pre-screening test for selecting participants for more invasive screening procedures, such as low-dose CT, appears to be rather modest compared to other cancer pre-screening tests. For example, the sensitivity and specificity of fecal immunochemical tests for hemoglobin, a widely used noninvasive test for pre-selecting participants for colonoscopy in colorectal cancer screening, are approximately 79% and 94%, respectively [23]. As can be seen from Appendix C and, Appendix D, such high levels of specificity, i.e., 94% or higher, were only achieved in women in a small number of countries, and they went along with sensitivities that were consistently below 40%. Sensitivities of at least 79% could be achieved only in very few countries with selected heavy smoking criteria, and only in combination with a specificity of 70% (for women in Greece) or lower. Pre-screening tests with such low specificity would commonly be considered unacceptable for population-based screening.

Our study also illustrates the striking variation in estimates of sensitivity and specificity of smoking as a pre-screening test for LC between populations. On first view, these results may appear to conflict with the common assumption that sensitivity and specificity, in contrast to positive and negative values, are intrinsic characteristics of screening tests that are invariant across populations in which they are applied. However, this assumption would only hold in case of homogeneity of cases across populations. With strongly varying smoking prevalences, the proportion of LC cases that is attributable to heavy smoking and hence the diagnostic performance parameters of heavy smoking will strongly vary across populations. Obviously, in a (hypothetical) population with 100% smoking prevalence, sensitivity and specificity of smoking would be 100% and 0%, respectively, whereas they would be 0% and 100%, respectively, in a (hypothetical) population with 0% smoking prevalence. The estimates derived in our study represent the sensitivity and specificity levels and their variation between sexes and countries encountered with the real life smoking histories and prevalences.

Given that smoking exposure is the currently most widely used pre-screening test for selecting participants for more invasive LC screening procedures [24], our results could serve as benchmarks for alternative or complementary noninvasive or minimally invasive, easy-to-implement pre-screening tests, such as more comprehensive risk factor scores, blood or breath tests [25]. For example, a number of studies have explored the potential of breath tests for LC detection and partly found similar combinations of sensitivity and specificity as those derived for the various smoking definitions in our study. For instance, Phillips and colleagues have demonstrated in a blinded validation setting sensitivities of 68.0 and 70.1% and specificities of 68.4 and 68.0% (analysis in two independent labs) for LC detection using volatile organic compounds in exhaled breath [18]. These estimates are comparable to pairs of smoking-based sensitivity and specificity derived for some of the countries in our analysis. With further improvement in biomarker technologies and discovery, biomarker-based algorithms may outperform smoking history-based risk stratification, and the performance of such risk stratification would be expected to be more consistent across countries with varying smoking prevalences. In addition, such tests would also not be affected by potential misclassification due to misreporting, which is a major concern for pre-screening based on smoking history [26]. However, potential benefits of including biomarkers in preselection for LC screening would have to be weighed against increased complexity and costs, and introduction of effective LC screening should not be delayed until biomarker-based approaches are fully validated and established. In the long run, the most promising approach for enhancing LC pre-screening might be to combine smoking-based pre-screening tests with complementary easy-to-implement noninvasive or minimally invasive tests, taking advantage of the information gained by both approaches.

In the interpretation of our study, a number of strengths and limitations need to be kept in mind. Strengths include the large sample sizes from a pooled analysis of case-control studies and from national surveys using comparable data collection methods from 27 European countries which enabled estimating relative risks by pack-year categories and smoking prevalences at high levels of precision across diverse populations.

However, our study also has limitations. First, relative risk estimates for pack-year categories used to derive sensitivity estimates of various screening criteria were available for current smokers as compared to never smokers only, and it was assumed that these relative risks also apply to former smokers who quit within 10 or 15 years. Second, the study populations included in the case-control studies from which the relative risk estimates were drawn were recruited in earlier decades (in the periods 1985–1996 in six studies, 1996–2002 in six studies and 1998–2005 in three studies) when the prevalence of daily smoking was higher as compared to now in most European countries [27]. Third, in the absence of available estimates of relative risks for preclinical, prevalent LC, our analysis was based on estimates of relative risk of incident, clinically manifest LC. This approximation could have had a major impact on the estimates of sensitivity and specificity in case of strong variation of sojourn time between pack-year categories which, though, seems to be unlikely. Fourth, we only addressed sensitivity and specificity as indicators of diagnostic performance of a pre-screening test. For the practice of screening, further parameters, such as the positive predictive value, which additionally depends on the prevalence of (undetected) LC among the target population, have to be considered. Like sensitivity of smoking as a pre-screening test for LC, the prevalence of (undetected) LC is also expected to be higher in populations with higher smoking prevalence which further underlines the fact that performance of heavy smoking criteria as pre-screening tests for LC will strongly vary between populations. Fifth, specificities were approximated by the proportion of individuals not meeting the pre-selection-criteria for LC screening among the target age groups of screening. This approximation neglects the proportion of people with prevalent LC among the target population of screening, which is expected to be very low and should therefore not have led to relevant distortions of specificity estimates. Sixth, our estimates are statistically derived from various data sources in the literature and should be validated in large-scale prospective studies.

## 4. Materials and Methods

### 4.1. Pre-Screening Criteria of Heavy Smoking Assessed in this Study

Different criteria used to select individuals for LC early detection/screening trials in Europe and recommendations for LC screening with low-dose CT from the U.S. were extracted from a recent world-wide review (Table 1, [24]). Eligibility for screening in different trials was either defined by pack-years (at least 20 [8,9,10,11] and at least 30 [3,5,6]), by smoking intensity in combination with the smoking duration [12,13,14], or by the individual risk model [7]. For former smokers, time since cessation was considered as additional criterion. While some variation in the target age range between trials was seen, the majority of participants were consistently in the age range from 50 to 74 years. We therefore chose this age range for our analysis. Among the eight definitions of eligibility for screening from European trials, four studies determined smoking history by pack-years [8,9,10,11] and these criteria, along with the NLST criteria from the U.S. trial were assessed in our analysis as pre-screening tests for which we calculated sensitivity and specificity in individual European countries with varying past and current smoking prevalences.

### 4.2. Strength of the Association between Smoking and Lung Cancer

Associations of smoking history with LC risk were extracted from a pooled analysis of eight case-control studies from Europe and one case-control study from Canada comprising 13,169 LC cases (81% males, 35% <60 years) and 16,010 sex—and age-matched controls [28]. In this study, an ever smoker was defined as an individual who had smoked at least one pack-year during their lifetime, and a current smoker was defined as an ever smoker who still smoked in the year of interview or in the year before. For our analysis, reported sex-specific odds ratios (ORs) for pack-year categories (>1–<20, 20–<30, 30–<40, 40–<50, 50–<60, and 60+) among current smokers as compared to never smokers were extracted (Table 2) and assumed to equally apply to former smokers who quit within the past 10 or 15 years for whom such data were not explicitly reported. Compared to never smokers, the ORs increased from 8.9 in the 1–<20 pack-years category to 47.7 in the ≥60 pack-years category in men, and from 3.5 to 25.7 among women. ORs were smaller among women as compared to men in each pack-year category.

### 4.3. Prevalence of Smoking in European Countries

The distribution of smoking histories in the respective age ranges within various European countries was derived from the Eurobarometer database (year 2017) [29]. This database, collected through face-to-face interviews, provided individual information on smoking intensity (cigarettes per day) and duration (derived from the age at starting and age at quitting when applicable) for both current and former smokers. To be consistent with the definition of smoking status in the pooled analysis of case-control studies used for risk estimates [28], ever smokers were defined as individuals with at least one pack-year smoking history and former smokers were individuals who quit at least one year ago (calculated as (age at interview)-(age of quitting smoking) >1). The minorities of individuals with insufficient information to derive pack-years or time of cessation (<15% of data among current and former smokers) were classified based on self-reported smoking status and these individuals were allocated to the respective pack-year categories according to the proportions observed among current and former smokers with this information. One country with a very high proportion of missing data (Portugal) was excluded from the analysis.

### 4.4. Statistical Analyses

We estimated key parameters of diagnostic performance, i.e., sensitivity and specificity, of various definitions of heavy smoking for LC detection among men and women in 27 European countries.

Sensitivity estimates were derived by combining proportions of smoking categories from the Eurobarometer database with the detailed results on the associations of smoking history with LC risk from the pooled analysis shown in Table 2, assuming that these associations for LC incidence equally apply to the prevalence of preclinical LC. Notations to calculate expected sensitivities of the various pack-year-based pre-screening criteria for detecting LC are shown in Table 3.

Using the notation and data sources shown in Table 3, sex—and country-specific expected sensitivities were derived for the European countries as follows:

The expected proportion of prevalent “smoking history positive” LC cases among the target populations for screening, denoted as LC_S_, was calculated as

(1) $${\mathrm{LC}}_{S}=\sum_{i}\left({\mathrm{RR}}_{i}*\mathrm{LN}*\left({\mathrm{PC}}_{i}+{\mathrm{PS}}_{i}\right)\right),$$

the expected total proportion of prevalent LC cases, denoted LC_T_, was calculated as

(2) $${\mathrm{LC}}_{T}=\mathrm{LN}*\mathrm{PN}+\sum_{j}\left({\mathrm{RR}}_{j}*\mathrm{LN}*\left({\mathrm{PC}}_{j}+{\mathrm{PS}}_{j}+{\mathrm{PL}}_{j}\right)\right)+\;\sum_{i}\left({\mathrm{RR}}_{i}*\mathrm{LN}*\left({\mathrm{PC}}_{i}+{\mathrm{PS}}_{i}+{\mathrm{PL}}_{I}\right)\right),$$

and the expected sensitivity, denoted SE, was calculated as

(3) $$\mathrm{SE}=\;\frac{{\mathrm{LC}}_{S}}{{\mathrm{LC}}_{T}}=\;\frac{\;\sum_{i}\left({\mathrm{RR}}_{i}*\;\left({\mathrm{PC}}_{i}+I\right)\right)\;}{\mathrm{PN}+\sum_{j}\left({\mathrm{RR}}_{j}*\left({\mathrm{PC}}_{j}+{\mathrm{PS}}_{j}+{\mathrm{PL}}_{j}\right)\right)+\;\sum_{i}\left({\mathrm{RR}}_{i}*\left({\mathrm{PC}}_{i}+{\mathrm{PS}}_{i}+{\mathrm{PI}}_{i}\right)\right)}$$

Note that this approach assumes that the impact of pack-years reported for current smokers by Pesch et al. [28] equally applies to former smokers who quit in the past 10 or 15 years (for whom it was not explicitly reported). This assumption appears justified and in agreement with screening recommendations given that only former smokers who quit relatively recently are recommended for LC screening and the same pack-year criteria as for current smokers are made for these recent quitters (see Table 1). The relative risk (RR) in the formulas was approximated by adjusted ORs reported by Pesch et al. [28]. A further assumption is that the relative risks estimated for incident clinically manifest (diagnosed) LC in epidemiological studies equally apply to the risk of preclinical (undiagnosed) prevalent LC which is the target of LC screening.

Specificities were approximated by the proportion of individuals not meeting the pre-selection criteria for LC screening among the target age groups of screening. This approximation is based on the plausible assumption of a low prevalence of preclinical but potentially CT-detectable LC at a certain point of time not only in the general population but even among heavy smokers.

## 5. Conclusions

Despite its limitations, our study may help to illustrate the order of magnitude of the diagnostic performance of established smoking-based pre-selection of participants for LC screening. Our results may furthermore illustrate the potential variation of diagnostic performance between various selection criteria and across populations with different smoking histories. Our results may thereby inform efforts to establish effective LC screening offers that are currently ongoing in many countries. In particular, they may help to choose between selection criteria and to stimulate further research towards enhanced instruments and criteria for preselecting those at highest risk for CT-based LC screening.

## Figures and Tables

**Table 1:** Various inclusion criteria used to select high-risk individuals for lung cancer screening in Europe and the U.S. derived from the data in reference [24].

Trial/Recommendation (Country)	Reference	Smoking History (Current and Former Smokers)	Restrictions for Former Smokers	Target Age
NLST (U.S.)	[3]	≥30 pack-years	quit within 15 years	55–74
U.S. Preventive Services Task Force ^1^ (U.S.)	[5]	≥30 pack-years	quit within 15 years	55–80
Centers for Medicare and Medicaid Services ^1^ (U.S.)	[6]	≥30 pack-years	quit within 15 years	55–77
MILD (Italy)	[8]	≥20 pack-years	quit within 10 years	49+
DANTE (Italy)	[9]	≥20 pack-years	quit within 10 years	60–74
ITALUNG (Italy)	[10]	≥20 pack-years	quit within 10 years	55–69
DLCST (Denmark)	[11]	≥20 pack-years	quit within 10 years	50–70
LUSI (Germany)	[13]	Either ≥15 cigarettes per day for at least 25 years or ≥10 cigarettes per day for at least 30 years	quit within 10 years	50–69
NELSON (Netherlands/Belgium)	[12]	Either ≥15 cigarettes per day for at least 25 years or ≥10 cigarettes per day for at least 30 years	quit within 10 years	50–75
DEPISCAN (France)	[14]	≥15 cigarettes per day for at least 20 years	quit within 15 years	50–74
UKLS (UK)	[7]	Individual risk for lung cancer assessed with the Liverpool Lung Project (LLP) risk model	n/a	50–75

^1^ Recommendation derived from the NLST trial [3].

**Table 2:** Relative risk of lung cancer in current smokers by pack-years derived from a pooled analysis of eight large European case-control studies and one Canadian study (reference [28]).

Smoking Status	Pack-Years	*N.* Cases	*N.* Controls	Odds Ratio (OR) (95% CI) ^1^
Men				
Never smokers	-	220	2883	1.0 (reference)
Current smokers	>1–<20	646	885	8.9 (7.4–10.6)
	20–<30	1213	880	17.1 (14.4–20.2)
	30–<40	1527	800	24.6 (20.8–29.0)
	40–<50	1324	582	32.4 (26.7–39.5)
	50–<60	770	243	46.3 (37.0–58.1)
	≥60	1245	414	47.7 (38.5–59.0)
Women				
Never smokers	-	609	1902	1.0 (reference)
Current smokers	>1–<20	330	305	3.5 (2.9–4.3)
	20–<30	304	160	7.3 (5.8–9.2)
	30–<40	291	97	12.9 (9.9–16.9)
	40–<50	204	61	14.0 (9.3–21.1)
	50–<60	129	31	17.9 (10.6–30.1)
	≥60	151	24	25.7 (14.5–45.5)

^1^ Estimated with logistic regression, conditional on study center, adjusted for age and smoking of other types of tobacco.

**Table 3:** Parameters used to derive expected sensitivity and specificity of the various smoking history criteria for detecting lung cancer in European populations.

Parameter	Notation	Source
Prevalence of lung cancer among never smokers ^1^	LN	
Relative risk for pack-year category i (j) above (below) threshold compared to never smokers	RR_i_ (RR_j_)	Pesch et al. [28]: Odds ratios in Table 2
Proportions of various subgroups in target age range of screening		Eurobarometer data base [29]
Never smokers	PN	
Current smokers in pack-year category i (j) above (below) threshold	PC_i_ (PC_j_)	
Short-term quitters in pack-year category i (j) above (below) threshold ^2^	PS_i_ (PS_j_)	
Long-term quitters in pack-year category i (j) above (below) threshold ^2^	PL_i_ (PL_j_)	

^1^ Unknown; this parameter is used to illustrate derivation of equations, but is not needed for final calculations of sensitivity and specificity. ^2^ Short- or long-term quitters are former smokers who quit within or beyond the time window used in the selection criterion for former smokers, respectively.

## Appendix A

Proportion of men in the target age range who meet study-specific criteria for lung cancer screening in European countries (sorted from highest to lowest prevalence of current smoking).

## Appendix B

Proportion of women in the target age range who meet study-specific criteria for lung cancer screening in European countries (sorted from highest to lowest prevalence of current smoking).

## Appendix C

Estimates of sensitivity and specificity of various smoking-based criteria as a pre-screening test for lung cancer in European countries among men (sorted from highest to lowest prevalence of current smoking).

## Appendix D

Estimates of sensitivity and specificity of various smoking-based criteria as a pre-screening test for lung cancer in European countries among women (sorted from highest to lowest prevalence of current smoking).
